# Effect of Sarcopenia on Sleep Disturbance in Patients with Chronic Liver Diseases

**DOI:** 10.3390/jcm8010016

**Published:** 2018-12-22

**Authors:** Hiroki Nishikawa, Hirayuki Enomoto, Kazunori Yoh, Yoshinori Iwata, Yoshiyuki Sakai, Kyohei Kishino, Naoto Ikeda, Tomoyuki Takashima, Nobuhiro Aizawa, Ryo Takata, Kunihiro Hasegawa, Noriko Ishii, Yukihisa Yuri, Takashi Nishimura, Hiroko Iijima, Shuhei Nishiguchi

**Affiliations:** Division of Hepatobiliary and Pancreatic disease, Department of Internal Medicine, Hyogo College of Medicine, Nishinomiya, Hyogo 663-8501, Japan; nishikawa_6392@yahoo.co.jp (H.N.); mm2wintwin@ybb.ne.jp (K.Y.); yo-iwata@hyo-med.ac.jp (Y.I.); sakai429@hyo-med.ac.jp (Y.S.); hcm.kyohei@gmail.com (K.K.); nikeneko@hyo-med.ac.jp (N.I.); tomo0204@yahoo.co.jp (T.T.); nobu23hiro@yahoo.co.jp (N.A.); chano_chano_rt@yahoo.co.jp (R.T.); hiro.red1230@gmail.com (K.H.); ishinori1985@yahoo.co.jp (N.I.); gyma27ijo04td@gmail.com (Y.Y.); tk-nishimura@hyo-med.ac.jp (T.N.); hiroko-i@hyo-med.ac.jp (H.I.); nishiguc@hyo-med.ac.jp (S.N.)

**Keywords:** chronic liver disease, sleep disturbance, pittsburgh sleep quality index, sarcopenia, grip strength

## Abstract

We sought to investigate the influence of sarcopenia as defined by muscle strength and skeletal muscle mass (SMM) on sleep disturbance as evaluated by the Japanese version of Pittsburgh Sleep Quality Index (PSQI-J) in chronic liver diseases (CLDs) (*n* = 419). Muscle strength and muscle mass were determined by grip strength (GS) and SMM using bioimpedance analysis. Patients were classified into four types: type A (*n* = 61); decreased GS and decreased SMM; type B (*n* = 45); decreased GS and non-decreased SMM; type C (*n* = 102); non-decreased GS and decreased SMM; and type D (*n* = 211); non-decreased GS and non-decreased SMM. Factors associated with PSQI-J score 6 or more were examined. PSQI-J score 0–5 (normal) was found in 253 (60.4%); 6–8 (mild) in 97 (23.2%); 9–11 (moderate) in 45 (10.7%) and 12 or more (severe) in 24 (5.7%). Univariate analysis identified three factors to be significantly associated with PSQI-J score 6 or more: presence of liver cirrhosis (LC) (*P* = 0.0132); our classification of type A; B; C and D (*P* < 0.0001) and serum albumin level (*P* = 0.0041). Multivariate analysis showed that type A (*P* = 0.0021) and type B (*P* = 0.0220) were significant independent factors. In conclusion, sarcopenia in CLDs appears to be closely associated with sleep disturbance mainly due to muscle strength decline.

## 1. Introduction

Sleep is essential for mental and physical health and there is growing interest in sleep worldwide. Liver cirrhosis (LC) patients frequently describe sleep problems and non-LC patients with chronic liver diseases (CLDs) are not exceptions [1,2,3,4,5,6,7,8,9,10,11]. The mechanisms of sleep disturbance in CLD patients are not well elucidated; however, they appear temporally to be associated with CLD itself [12]. Persistent elevated melatonin levels due to impaired melatonin metabolism in LC patients can lead to disrupted circadian rhythms [13,14]. Sleep disturbances negatively impact innate immunity and are commonly associated with neurocognitive alterations in CLD patients regardless of the severity of liver fibrosis [2,15]. They include difficulty with falling asleep, fragmented sleep in night-time and increase in daytime somnolence, and have been suggested to adversely affect the quality of life (QOL) in CLD patients [8,15,16,17]. Thus, examining factors associated with sleep disturbances in CLD patients is clinically meaningful. Currently, one of extensively used and well validated patient-reported sleep questionnaires is Pittsburgh Sleep Quality Index (PSQI) [18,19,20].

On the other hand, sarcopenia is a disease entity combining poor muscle function and diminished skeletal muscle mass (SMM), which results in frailty, cachexia, and osteoporosis [21,22,23,24,25,26,27,28,29,30,31,32]. It can also be associated with worse patient QOL, prognosis and higher health care costs in various diseases including CLDs [22,24,26]. Increase of SMM is generally observed until 20 years and SMM reaches a peak between 20 and 50 years. Thereafter, it decreases by approximately 1% after the age of 50 years because of qualitative and quantitative changes in muscle fibers [33]. Sarcopenia is the main component of poor nutrition and is primarily responsible for the adverse clinical consequences seen in CLD patients [22,24,26]. In 2016, the Japanese Society of Hepatology (JSH) established the original criteria for liver diseases-related sarcopenia based on the Asian criteria for sarcopenia [21,25]. The JSH criteria adopts grip strength (GS) for the evaluation of muscle strength and bioimpedance analysis (BIA) and/or computed tomography for the evaluation of muscle mass, while unlike the Asian criteria for sarcopenia, there is no age restriction (i.e., 65 years old or more) in the JSH criteria for the evaluation of sarcopenia since CLD patients can have diminished SMM owing to impaired protein synthesis regardless of age [21]. 

However, as far as we are aware, data for the relevance between sarcopenia and sleep disturbances in CLD patients remain limited. In the current study, we sought to investigate the influence of sarcopenia on sleep disturbance as evaluated by the Japanese version of PSQI (PSQI-J) in patients with CLDs [20]. 

## 2. Patients and Methods

### 2.1. Patients

This study was a single-center retrospective study. A total of 440 CLD patients with data for GS, SMM using BIA and the PSQI available were admitted to our hospital between November 2013 and August 2018. Fifteen patients with underlying diseases such as overt hepatic encephalopathy, advanced malignancies, severe inflammatory diseases, or severe psychiatric diseases were excluded because they potentially affected the interpretation of PSQI score. As overestimates could occur for the calculation of skeletal muscle mass index (SMI) using BIA in patients with massive ascites, 6 subjects with severe ascites were also excluded from the study [22]. Four-hundred and 19 patients were thus analyzed. There were 156 LC patients (37.2%). In 71 LC patients, LC was determined by histological and imaging findings, and in the remaining 85 patients, LC was determined by imaging findings alone. 

### 2.2. Questionnaire Survey

Sleep quality was assessed using PSQI-J as a screening tool for sleep disturbance [18,19,20]. The questionnaire consists of a total of 10 queries that form 7 categories: (i) subjective sleep quality, (ii) sleep latency, (iii) sleep duration, (iv) habitual sleep efficiency, (v) sleep disorders, (vi) use of sleep medications and (vii) daytime disturbance. Each category is rated on a scale of 0 to 3, and the sum of PSQI-J scores for all categories is 21 points. Higher PSQI-J scores suggest a poorer sleep quality. Favorable sensitivity and specificity were observed when the sum of PSQI-J scores exceeded 6 points [19]. Our study subjects were categorized as normal (PSQI-J score, 0–5), and the severity of sleep disturbance as mild (PSQI-J score, 6–8), moderate (PSQI-J score, 9–11) and severe (PSQI-J score, 12 or more) [18,19,20]. 

### 2.3. Measurement of GS and SMI and our Study

GS was measured according to the current guidelines [21]. Two measurements of GS were performed on both the left and right sides. The better measurement on each side is used, and GS was calculated as the mean of these values. SMI was defined as “appendicular SMM/(height (m))^2^” using BIA. According to the current guidelines, patients with decreased GS (D-GS) were defined as those with GS < 26 kg for men and <18 kg for women. Similarly, patients with decreased SMM (D-SMM) were defined as those with SMI < 7.0 kg/m^2^ for men and <5.7 kg/m^2^ for women [21]. Again, the JSH criteria for sarcopenia in liver disease determines sarcopenia based on muscle strength and muscle mass irrespective of age considering the possibility that younger advanced LC patients suffer from sarcopenia [21]. In male, patients with GS < 26 kg and SMI < 7.0 kg/m^2^ were classified as type A (sarcopenia), those with GS < 26 kg and SMI ≥ 7.0 kg/m^2^ as type B, those with GS ≥ 26 kg and SMI < 7.0 kg/m^2^ as type C, and those with GS ≥26 kg and SMI ≥ 7.0 kg/m^2^ as type D. In female, patients with GS < 18 kg and SMI < 5.7 kg/m^2^ were classified as type A (sarcopenia), those with GS < 18 kg and SMI ≥5.7 kg/m^2^ as type B, those with GS ≥ 18 kg and SMI < 5.7 kg/m^2^ as type C, and those with GS ≥ 18 kg and SMI ≥ 5.7 kg/m^2^ as type D (Table 1).

First, the impacts of GS and SMM on sleep disturbance as assessed by PSQI-J were examined for all cases and several subgroups according to the LC status, gender, and age. PSQI-J scores among 4 types (type A, B, C and D) were also compared. Subsequently, factors associated with PSQI-J score 6 or more (mild, moderate, or severe sleep disturbance) were studied using univariate and multivariate analyses. We received the ethical approval from ethics committee of our hospital (approval no, 2296). The protocol in the study strictly observed all regulations of the Declaration of Helsinki. 

### 2.4. Statistical Considerations

Comparisons between groups were performed for all cases and several subgroups according to the LC status, gender, and age. As for continuous parameters, Student’s *t* test, Mann-Whitney U test, analysis of variance or Kruskal-Wallis test were employed to assess group difference, as applicable. In categorical parameters, Fisher’s exact tests or Pearson χ^2^ test was employed to assess group difference, as applicable. Factors with *P* < 0.1 linked to PSQI-J score 6 or more in the univariate analysis were subjected to the multivariate logistic regression analysis to identify candidate parameters. Unless otherwise mentioned, data were indicated as median value (range). The threshold for statistical significance was set at *P* < 0.05. The JMP 13.2 (SAS Institute Inc., Cary, NC, USA) was employed to carry out statistical analysis.

## 3. Results

### 3.1. Patient Baseline Characteristics

**Figure 1:**
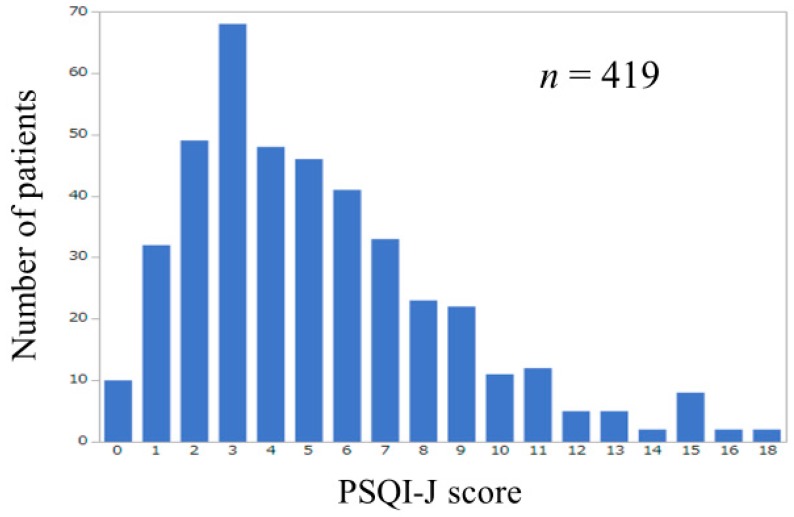
Number of patients according to PSQI-J score (*n* = 419).

Baseline characteristics in our study (*n* = 419, 200 men and 219 women, median age = 64 years) were presented in Table 2. The number of patients according to PSQI-J score was shown in Figure 1. The median (range) PSQI-J score was 5 (0–18). PSQI-J score 0–5 (normal) was found in 253 (60.4%), 6–8 (mild) in 97 (23.2%), 9–11 (moderate) in 45 (10.7%) and 12 or more (severe) in 24 (5.7%) [11]. Sleep disturbance with PSQI-J score 6 or more was thus observed in 166 patients (39.6%). The median (range) GS and SMI in male patients were 34.3 kg (6.6–57.8 kg) and 7.5 kg/m^2^ (5.2–11.0 kg/m^2^) and those in female patients were 20.1 kg (5.9–36.1 kg) and 5.9 kg/m^2^ (3.9–8.1 kg/m^2^). Sarcopenia (i.e., type A) as defined by the JSH criteria was observed in 27 male patients (13.5%) and 34 female patients (15.5%) [21]. Among type A, B, C and D, overall differences were noted with statistical significance in age, gender, presence of LC, body mass index (BMI), serum albumin, prothrombin time, platelet count, total cholesterol, estimated glomerular filtration rate and PSQI-J score (Table 2). The median (range) PSQI-J score in LC patients (5 (0–18)) was significantly higher than that in non-LC patients (4 (0–15)) (*P* = 0.0046), suggesting the poorer sleep quality in LC patients compared with non-LC patients. The median (range) PSQI-J score in male patients (4 (0–16)) was significantly lower than that in female patients (5 (0–18)) (*P* = 0.0420). The median (range) PSQI-J score in patients aged 65 years or more (4.5 (0–18)) was similar to that in patients aged less than 65 years (5 (0–18)) (*P* = 0.5118).

### 3.2. Influence of GS and SMM on PSQI-J for All Cases (n = 419)

**Figure 2:**
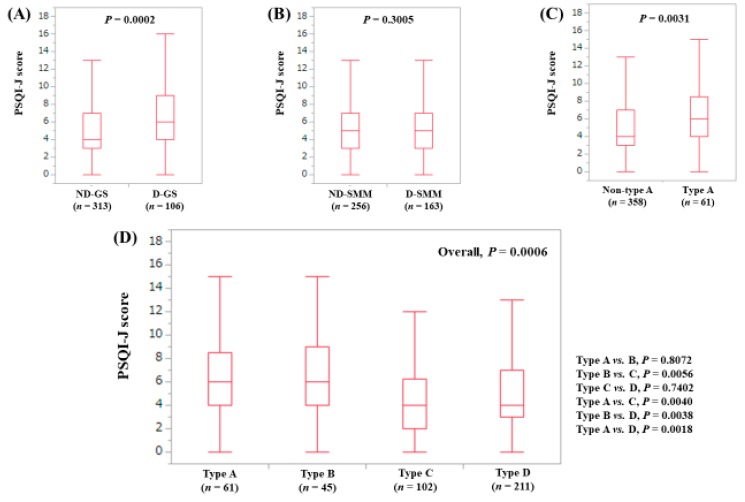
PSQI-J score stratified by GS and SMM for all cases (*n* = 419). (**A**) Non-decreased GS vs. decreased GS. (**B**) Non-decreased SMM vs. decreased SMM. (**C**) Non-type A (type B, C and D) vs. type A. (**D**) Comparison among four types (type A, B, C and D).

The median (range) PSQI-J score in the D-GS (6 (0–18), *n* = 106) was significantly higher than that in the non-decreased GS (ND-GS) (4 (0–18), *n* = 313) (*P* = 0.0002), (Figure 2A). The median (range) PSQI-J score in the D-SMM (5 (0–18), *n* = 163) was similar to that in the non-decreased SMM (ND-SMM) (5 (0–18), *n* = 256) (*P* = 0.3005), (Figure 2B). The median (range) PSQI-J score in type A (sarcopenia) (6 (0–18), *n* = 61) was significantly higher than that in non-type A (i.e., type B, C and D) (5 (0–18), *n* = 358) (*P* = 0.0031), (Figure 2C). In comparison among four types, the overall difference was observed with significance (*P* = 0.0006), (Figure 2D).

### 3.3. Stratified Analysis 1: Influence of GS and SMM on PSQI-J for LC Patients (n = 156)

**Figure 3:**
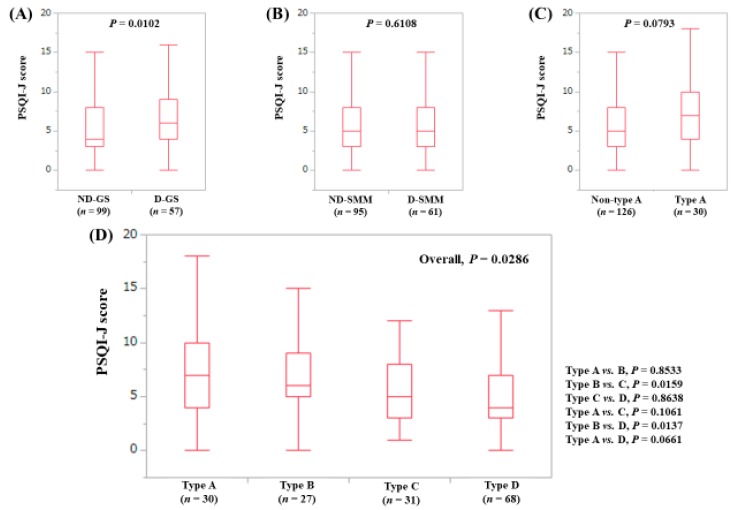
PSQI-J score stratified by GS and SMM for LC cases (*n* = 156). (**A**) Non-decreased GS vs. decreased GS. (**B**) Non-decreased SMM vs. decreased SMM. (**C**) Non-type A (type B, C and D) vs. type A. (**D**) Comparison among four types (type A, B, C and D).

The median (range) PSQI-J score in the D-GS (6 (0–18), *n* = 57) was significantly higher than that in the ND-GS (4 (0–18), *n* = 99) (*P* = 0.0102), (Figure 3A). The median (range) PSQI-J score in the D-SMM (5 (0–18), *n* = 61) was similar to that in the ND-SMM (5 (0–18), *n* = 95) (*P* = 0.6108), (Figure 3B). The median (range) PSQI-J score in type A (7 (0–18), *n* = 30) tended to be significantly higher compared with non-type A (5 (0–18), *n* = 126) (*P* = 0.0793), (Figure 3C). In comparison among four types, the overall difference was noted with significance (*P* = 0.0286), (Figure 3D).

### 3.4. Stratified Analysis 2: Influence of GS and SMM on PSQI-J for Non-LC Patients (n = 263)

**Figure 4:**
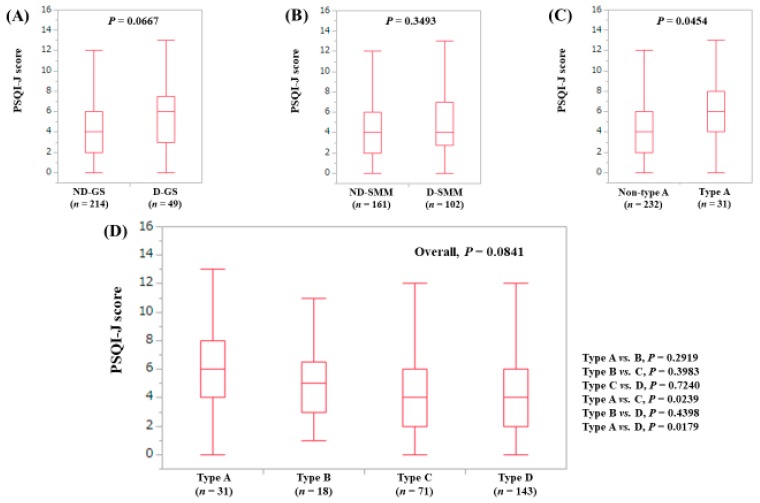
PSQI-J score stratified by GS and SMM for non-LC cases (*n* = 263). (**A**) Non-decreased GS vs. decreased GS. (**B**) Non-decreased SMM vs. decreased SMM. (**C**) Non-type A (type B, C and D) vs. type A. (**D**) Comparison among four types (type A, B, C and D).

The median (range) PSQI-J score in the D-GS (6 (0–13), *n* = 49) had a tendency for significance compared with the ND-GS (4 (0–15), *n* = 214) (*P* = 0.0667), (Figure 4A). The median (range) PSQI-J score in the D-SMM (4 (0–15), *n* = 102) was equal to that in the ND-SMM (4 (0–15), *n* = 161) (*P* = 0.3493), (Figure 4B). The median (range) PSQI-J score in type A (6 (0–13), *n* = 31) was significantly higher compared with non-type A (4 (0–15), *n* = 232) (*P* = 0.0454), (Figure 4C). In comparison among four types, the overall difference was noted with a trend for significance (*P* = 0.0841), (Figure 4D).

### 3.5. Stratified Analysis 3: Influence of GS and SMM on PSQI-J for Male Patients (n = 200)

**Figure 5:**
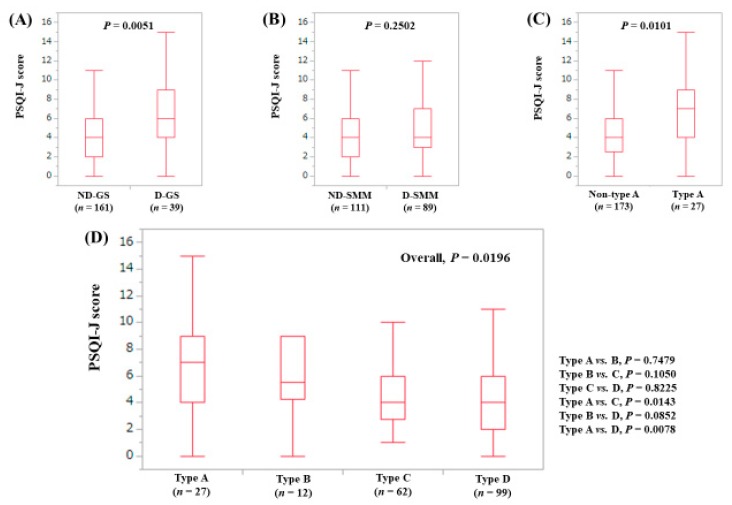
PSQI-J score stratified by GS and SMM for male cases (*n* = 200). (**A**) Non-decreased GS vs. decreased GS. (**B**) Non-decreased SMM vs. decreased SMM. (**C**) Non-type A (type B, C and D) vs. type A. (**D**) Comparison among four types (type A, B, C and D).

The median (range) PSQI-J score in the D-GS (6 (0–15), *n* = 39) was significantly higher compared with the ND-GS (4 (0–16), *n* = 161) (*P* = 0.0051), (Figure 5A). The median (range) PSQI-J score in the D-SMM (4 (0–16), *n* = 89) was identical to that in the ND-SMM (4 (0–15), *n* = 111) (*P* = 0.2502), (Figure 5B). The median (range) PSQI-J score in type A (7 (0–15), *n* = 27) was significantly higher than that in non-type A (4 (0–16), *n* = 173) (*P* = 0.0101), (Figure 5C). In comparison among four types, the overall difference was identified with significance (*P* = 0.0196), (Figure 5D).

### 3.6. Stratified Analysis 4: Influence of GS and SMM on PSQI-J for Female Patients (n = 219)

**Figure 6:**
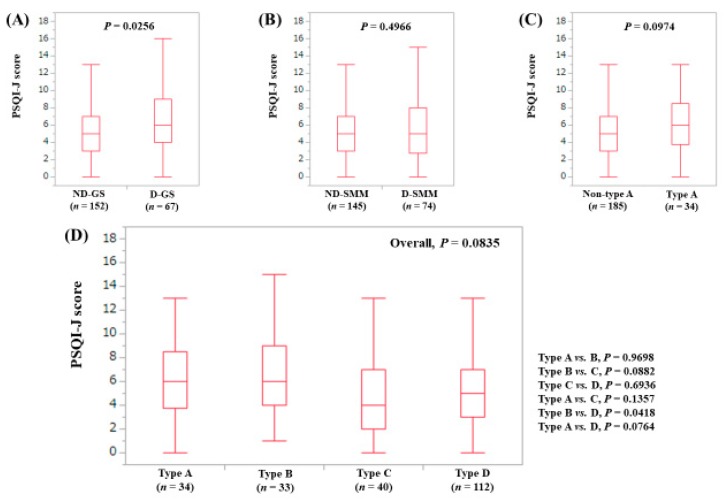
PSQI-J score stratified by GS and SMM for female cases (*n* = 219). (**A**) Non-decreased GS vs. decreased GS. (**B**) Non-decreased SMM vs. decreased SMM. (**C**) Non-type A (type B, C and D) vs. type A. (**D**) Comparison among four types (type A, B, C and D).

The median (range) PSQI-J score in the D-GS (6 (0–18), *n* = 67) was significantly higher compared with the ND-GS (5 (0–18), *n* = 152) (*P* = 0.0256), (Figure 6A). The median (range) PSQI-J score in the D-SMM (5 (0–18), *n* = 74) was similar to that in the ND-SMM (5 (0–18), *n* = 145) (*P* = 0.4966), (Figure 6B). The median (range) PSQI-J score in type A (6 (0–18), *n* = 34) had a trend for significance compared to non-type A (5 (0–18), *n* = 185) (*P* = 0.0974), (Figure 6C). In comparison among four types, the overall difference was noted with a tendency for significance (*P* = 0.0835), (Figure 6D).

### 3.7. Stratified Analysis 5: Influence of GS and SMM on PSQI-J for Patients Aged 65 Years or More (n = 202)

**Figure 7:**
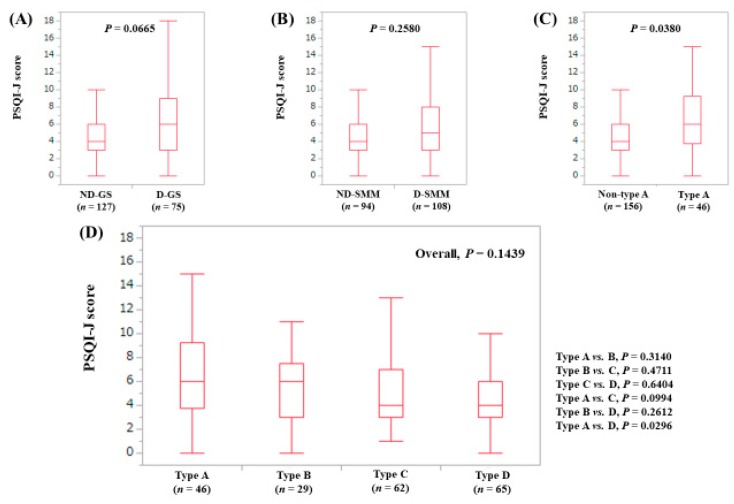
PSQI-J score stratified by GS and SMM for patients aged 65 years or more (*n* = 202). (**A**) Non-decreased GS vs. decreased GS. (**B**) Non-decreased SMM vs. decreased SMM. (**C**) Non-type A (type B, C and D) vs. type A. (**D**) Comparison among four types (type A, B, C and D).

The median (range) PSQI-J score in the D-GS (6 (0–18), *n* = 75) tended to be significantly higher than that in the ND-GS (4 (0–16), *n* = 127) (*P* = 0.0665), (Figure 7A). The median (range) PSQI-J score in D-SMM (5 (0–18), *n* = 108) was equal to that in the ND-SMM (4 (0–15), *n* = 94) (*P* = 0.2580), (Figure 7B). The median (range) PSQI-J score in type A (6 (0–18), *n* = 46) was significantly higher compared with non-type A (4 (0–16), *n* = 156) (*P* = 0.0380), (Figure 7C). In comparison among four types, the overall difference was not significant (*P* = 0.1439), (Figure 7D).

### 3.8. Stratified Analysis 6: Influence of GS and SMM on PSQI-J for Patients Aged Less Than 65 Years (n = 217)

**Figure 8:**
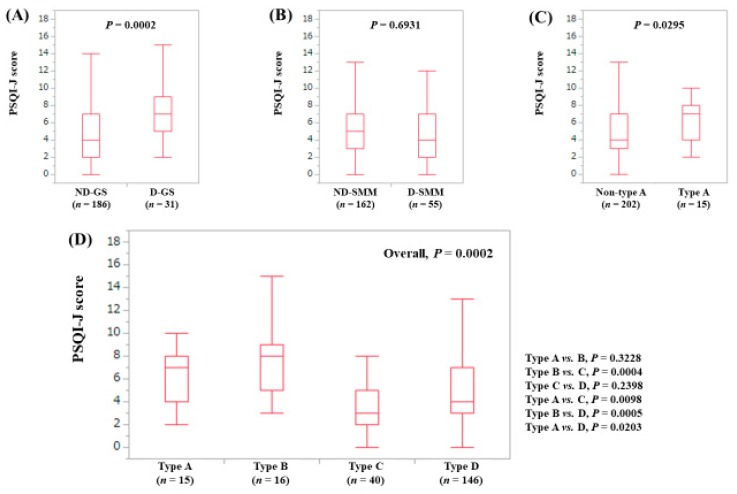
PSQI-J score stratified by GS and SMM for patients less than 65 years (*n* = 217). (**A**) Non-decreased GS vs. decreased GS. (**B**) Non-decreased SMM vs. decreased SMM. (**C**) Non-type A (type B, C and D) vs. type A. (**D**) Comparison among four types (type A, B, C and D).

The median (range) PSQI-J score in the D-GS (7 (2–16), *n* = 31) was significantly higher than that in the ND-GS (4 (0–18), *n* = 186) (*P* = 0.0002). (Figure 8A) The median (range) PSQI-J score in the D-SMM (4 (0–16), *n* = 55) was identical to that in the ND-SMM (5 (0–18), *n* = 162) (*P* = 0.6931). (Figure 8B) The median (range) PSQI-J score in type A (7 (2–16), *n* = 15) was significantly higher compared with non-type A (4 (0–18), *n* = 202) (*P* = 0.0295). (Figure 8C) In comparison among four types, the overall difference was noted with significance (*P* = 0.0002). (Figure 8D).

### 3.9. Univariate and Multivariate Analyses of Factors Associated with PSQI-J Score 6 or More

Univariate analysis identified three factors to be significantly associated with PSQI-J score 6 or more: presence of LC (*P* = 0.0132), our classification of type A, B, C and D (*P* < 0.0001) and serum albumin level (*P* = 0.0041), while gender tended to be significant (*P* = 0.0721), (Table 3). Multivariate analysis for the four factors showed that type A (*P* = 0.0021) and type B (*P* = 0.0220) were significant factors linked to PSQI-J score 6 or more (Table 4). Hazard ratios (HRs) and 95% confidence intervals for these factors are listed in Table 4. 

## 4. Discussion

Sleep disruption entails a heavy burden, and adversely impacts patient QOL, mood alterations, and possibly fatigue, which can be related to unfavorable clinical outcomes [10,11,15]. To our knowledge, this is the first study demonstrating the relevance between sleep disturbance and sarcopenia in CLD patients. To clarify these clinical questions is of importance because liver disease-related sarcopenia has been gaining much caution these days due to its significant correlation with QOL and prognosis [21,22,23,24,25,26,27,28,29,30,31].

In our results, compared with ND-GS patients, D-GS patients had higher PSQI-J scores with statistical significance or near significance for all analyses, while PSQI-J scores in patients with D-SMM were identical to those with ND-SMM for all analyses. Additionally, type A (sarcopenia) and type B (D-GS and ND-SMM) were independent predictors linked to PSQI-J score 6 or more in the multivariate analysis (HR = 0.393 in the type A and HR = 0.453 in the type B (type D as a reference)). These results denoted that sarcopenia in CLDs was closely associated with sleep disturbance and this was mainly influenced by muscle strength decline and SMM itself did not affect sleep quality. In this respect, our study results appear to shed some insights on the better understanding of sarcopenia and sleep disturbance in CLD patients. For sarcopenic CLD patients with sleep disturbance, physical exercise may be useful [34]. Recent reports trend towards a beneficial effect of physical exercise with improvement in QOL [34]. 

CLD patients are aging these days in our country and aging itself can cause sleep disturbance in older adults [35,36,37,38]. Serum melatonin levels, which regulate circadian rhythms such as the sleep-wake rhythm, vary considerably with aging [14]. Our data of median ages in the types of A, B, C, and D were 71, 69, 67 and 58 years, respectively, indicating the impact of aging on sarcopenia. On the other hand, the PSQI-J score in patients aged 65 years or more was similar to that in patients aged less than 65 years (*P* = 0.5118) in this study. Type A and B patients tended to be older than other types and type A and B were independent predictors associated with PSQI-J score 6 or more. While type A and B patients had poorer liver function as reflected by lower serum albumin levels. In addition, in our univariate analysis, presence of LC and serum albumin were significant factors linked to PSQI-J score 6 or more, whereas age was not. In view of these results, impaired protein synthesis due to poor liver function rather than aging itself appears to cause sleep disturbances in CLD patients. 

In our data, the median (range) PSQI-J score in LC patients (5 (0–18)) was significantly higher vs. non-LC patients (4 (0–15), *P* = 0.0046) and the proportion of PSQI-J score 6 or more in LC and non-LC patients were 47.4% (74/156) and 35.0% (92/263) (*P* = 0.0132). A recent study reported that 81% in LC patients had disturbed sleep with PSQI score 6 or more and sleep disruptions were associated with muscle cramps, increase in daytime somnolence, and poorer QOL [11]. Although the reasons for this discrepancy (i.e., 47.4% in our LC patients vs. 81% [11] in the frequency of disturbed sleep) are unclear, clinicians should be fully aware that sleep disturbance represents a pivotal unmet medical need in CLD patients [11]. On the other hand, the median (range) PSQI-J score in male (4 (0–16)) was significantly lower vs. female (5 (0–18)) (*P* = 0.0420), indicating poorer sleep quality in female patients. While gender was not an independent factor associated with PSQI-J 6 or more, gender difference for sleep disturbance cannot be ignored to provide CLD patients with individualized disease management [39].

In our subgroup analysis in patients aged less than 65 years (*n* = 217), overall difference in comparison among four types (type A, B, C and D) was noted with the strongest *P* value (*P* = 0.0002). Of these 217 patients, 78 patients (35.9%) and 15 patients (6.9%) had PSQI-J score 6 or more and sarcopenia. Primary sarcopenia is an aging-related sarcopenia and secondary sarcopenia is a disease-specific sarcopenia [21]. As mentioned above, the current JSH criteria for sarcopenia in liver disease determines sarcopenia based on muscle strength and muscle mass irrespective of age. Our study data may support the validity of the current JSH guidelines for sarcopenia. 

Obesity, insulin resistance and poor nutrition are reported to be associated with sleep disruptions [9,40,41]. Bernsmeier C, et al demonstrated that non-alcoholic fatty liver disease patients had shortened sleep duration, prolonged time to fall asleep, and poor sleep quality [9]. In our univariate analyses, serum albumin was significant linked to JSQI-J score 6 or more, while total cholesterol level, BMI and HbA1c were not. Additionally, in the subgroup analyses according to liver disease etiologies (hepatis B virus, hepatitis C virus and non-B and non-C), total cholesterol level, BMI and HbA1c were not significant linked to PSQI-J score 6 or more (data not shown). The reasons for these remain uncertain and further investigations will be necessary to confirm these results. 

Our study was associated with several limitations. Firstly, the study was a single-center observational study with a retrospective nature. Secondly, PSQI is a subjective assessment method for sleep disturbance and objective assessment for sleep such as actigraphy was not performed in this study [4]. Thirdly, GS can vary depending on patient daily life activities. Fourthly, patients with massive ascites or overt hepatic encephalopathy who are potentially involved in liver disease-related sarcopenia were excluded due to the lack of reliability in BIA or patient-administered questionnaire, leading to bias. Fifthly, data for alcohol use are lacking and patients with minimal hepatic encephalopathy which potentially affects PSQI-J score may be included in our study subjects, also creating bias [42]. Finally, it was unclear as to whether sarcopenia caused sleep disturbance or whether sleep disturbance caused sarcopenia in this study. Caution should be therefore exercised for the interpretation of our data. Nevertheless, our study results indicated that sarcopenia in CLDs was closely associated with sleep disturbance mainly due to muscle strength decline. 

## 5. Conclusions

Sarcopenia can be an independent predictor for sleep disturbance in patients with CLDs. Clinicians should be aware of the presence of sarcopenia in CLD patients with sleep disturbance.

## Figures and Tables

**Table 1:** Our type classification stratified by grip strength (GS) and skeletal muscle mass index (SMI).

	Male		Female	
	GS < 26 kg	SMI < 7.0 kg/m^2^	GS < 18 kg	SMI < 5.7 kg/m^2^
Type A	Yes	Yes	Yes	Yes
Type B	Yes	No	Yes	No
Type C	No	Yes	No	Yes
Type D	No	No	No	No

**Table 2:** Baseline characteristics.

Variables	All Cases (*n* = 419)	Type A (*n* = 61)	Type B (*n* = 45)	Type C (*n* = 102)	Type D (*n* = 211)	Overall (Type A, B, C and D) *P* Value
Age (years)	64 (25–90)	71 (25–90)	69 (33–83)	67 (29–84)	58 (26–90)	<0.0001
Gender, male/female	200/219	27/34	12/33	62/40	99/112	0.0016
HBV/HCV/HBV and HCV/NBNC	61/259/8/91	5/42/2/12	7/28/0/10	15/67/3/17	34/122/3/52	0.5540
Presence of LC, yes/no	156/263	30/31	27/18	31/71	68/143	0.0004
Body mass index (kg/m^2^)	22.6 (14.8–41.4)	21.3 (17.2–26.9)	25.5 (19.8–32.8)	20.6 (16.0–28.3)	24.1 (14.8–41.4)	<0.0001
Total bilirubin (mg/dL)	0.8 (0.3–5.6)	0.7 (0.4–2.7)	0.85 (0.3–4.0)	0.9 (0.3–2.4)	0.8 (0.3–5.6)	0.1415
Serum albumin (g/dL)	4.2 (1.8–5.2)	4.1 (2.4–4.7)	4.1 (2.5–5.2)	4.3 (2.3–5.0)	4.2 (1.8–4.9)	<0.0001
Prothrombin time (%)	88.7 (23.0–122.9)	85.3 (46.5–122.5)	80.4 (44.6–110.1)	90.0 (23.0–120.2)	89.8 (28.3–122.9)	0.0002
Platelet count (×10^4^/mm^3^)	16.8 (1.4–42.2)	15.3 (2.8–39.7)	13.1 (3.6–30.0)	15.3 (4.1–33.0)	17.8 (1.4–42.2)	0.0012
Total cholesterol (mg/dL)	177.5 (80–420)	175 (80–420)	158 (88–244)	182 (99–290)	181 (90–292)	0.0080
AST (IU/L)	28 (10–222)	29 (10–191)	34 (15–133)	27 (11–143)	27 (12–222)	0.2732
ALT (IU/L)	24 (5–297)	27 (5–263)	28 (5–213)	23 (5–188)	24 (8–297)	0.8079
ALP (IU/L)	239 (91–5065)	253.5 (121–5065)	249 (95–579)	231 (112–650)	234 (91–1206)	0.1702
GGT (IU/L)	28 (7–542)	26 (8–462)	37 (11–311)	26.5 (10–386)	27 (7–542)	0.8156
eGFR (mL/min/1.73 m^2^)	83 (5–162)	80 (87–141)	83.5 (31–146)	83 (5–136)	83.5 (34–162)	0.0497
HbA1c (NGSP)	5.7 (3.7–10.4)	5.6 (4.7–8.8)	5.6 (4.1–8.2)	5.7 (3.8–10.1)	5.6 (3.7–10.4)	0.9597
Serum sodium (mmol/L)	140 (124–148)	140 (124–144)	140 (130–146)	140 (131–148)	140 (126–144)	0.2000
PSQI-J score	5 (0–18)	6 (0–18)	6 (0–15)	4 (0–16)	4 (0–18)	0.0006

Data are expressed as median value (range). HBV; hepatitis B virus, HCV; hepatitis C virus, NBNC; non-B and non-C, LC; liver cirrhosis, AST; aspartate aminotransferase, ALT; alanine aminotransferase, ALP; alkaline phosphatase, GGT; gamma-glutamyltransferase, eGFR; estimated glomerular filtration rate, NSGP; National Glycohemoglobin Standardization Program, PSQI-J; Japanese version of Pittsburgh Sleep Quality Index.

**Table 3:** Univariate analyses of factors linked to PSQI-J score 6 or more.

Variables	PSQI-J Score 6 or More (*n* = 166)	PSQI-J Score Less than 6 (*n* = 253)	*P* Value
Age (years)	65 (26–90)	62 (25–90)	0.2326
Gender, male/female	70/96	130/123	0.0721
HBV/HCV/HBV and HCV/NBNC	21/106/3/36	40/153/5/55	0.8294
Body mass index (kg/m^2^)	22.9 (16.0–41.4)	22.5 (14.8–36.5)	0.1589
Presence of LC, yes/no	74/92	82/171	0.0132
Type, A/B/C/D	36/26/32/72	25/19/70/139	<0.0001
Total bilirubin (mg/dL)	0.8 (0.3–5.6)	0.8 (0.3–4.0)	0.6316
Serum albumin (g/dL)	4.1 (1.8–5.0)	4.2 (2.3–5.2)	0.0041
Prothrombin time (%)	87.5 (38.4–117.6)	90.0 (23.0–122.9)	0.1593
Platelet count (×10^4^/mm^3^)	16.5 (2.8–42.2)	16.9 (1.4–37.6)	0.5222
AST (IU/L)	29.5 (12–191)	28 (10–222)	0.6180
ALT (IU/L)	25 (5–297)	24 (5–213)	0.5807
ALP (IU/L)	235 (105–5065)	244 (91–1206)	0.9472
GGT (IU/L)	28.5 (8–462)	27 (7–542)	0.5677
Total cholesterol (mg/dL)	175.5 (80–420)	179.5 (88–290)	0.3166
eGFR (mL/min/1.73m^2^)	83 (83–162)	83 (5–147)	0.9792
Serum sodium (mmol/L)	140 (124–148)	140 (131–146)	0.6521
HbA1c (NGSP)	5.7 (3.7–10.1)	5.7 (3.8–10.4)	0.7734

PSQI-J: Japanese version of Pittsburgh Sleep Quality Index, HBV: hepatitis B virus, HCV: hepatitis C virus, NBNC: non-B and non-C, LC: liver cirrhosis, AST: aspartate aminotransferase, ALT: alanine aminotransferase, ALP: alkaline phosphatase, GGT: gamma-glutamyltransferase, eGFR: estimated glomerular filtration rate, NSGP: National Glycohemoglobin Standardization Program PSQI-J: Japanese version of Pittsburgh Sleep Quality Index

**Table 4:** Multivariate analyses of factors linked to PSQI-J score 6 or more.

	Multivariate Analysis		
	Hazard Ratio	95% CI	*P* Value
Presence of LC			
Yes	0.796	0.483–1.310	0.3704
No	1.000	Reference	
Gender			
Male	1.369	0.900–2.082	0.1412
Female	1.000	Reference	
Serum albumin (per 1.0 g/dL)	1.320	0.831–2.095	0.2390
Type			
A (D-GS and D-SMM)	0.393	0.217–0.713	0.0021
B (D-GS and ND-SMM)	0.453	0.230–0.892	0.0220
C (ND-GS and D-SMM)	1.020	0.609–1.710	0.9386
D (ND-GS and ND-SMM)	1.000	Reference	

PSQI-J: Japanese version of Pittsburgh Sleep Quality Index, LC: liver cirrhosis, D-GS: decreased grip strength, D-SMM: decreased skeletal muscle mass, ND-GS: non-decreased grip strength, ND-SMM: non-decreased skeletal muscle mass, CI: confidence interval
